# Perspectives of Patient Handover among Paramedics and Emergency Department Members; a Qualitative Study

**Published:** 2017-08-17

**Authors:** Majid Najafi Kalyani, Zheila Fereidouni, Raheleh Sabet Sarvestani, Zahra Hadian Shirazi, Ali Taghinezhad

**Affiliations:** 1 **Nursing Department, Fasa University of Medical Sciences, Fasa, Iran.**; 2 **N** **ursing Department, Shiraz University of Medical Sciences, Fars, Iran.**; 3 English language Department, Fasa University of Medical Sciences, Fasa, Iran.

**Keywords:** Allied health personnel, patient handoff, patient safety, emergency medical services, emergency service, hospital

## Abstract

**Introduction:**

Improving patient handover is currently considered as a patient safety goal and one of the top five WHO priorities. So, the aim of this study was to explore the perspectives of patient handover among paramedics and emergency department staff.

**Methods:**

This is a descriptive exploratory study with a qualitative content analysis approach. Twenty five paramedics and emergency department staff were selected through purposeful sampling. The data were collected through semi-structured interviews in 2015 and Qualitative Content Analysis was used to analyze the data.

**Result:**

One main theme and two major categories emerged through the data analysis. In general, data analysis indicated that patient handover is a sophisticated process, which is an encounter between two separate peninsulas with different extrinsic (different environments and different equipment) and intrinsic factors (different manpower and different expectations).

**Conclusion:**

Designing an appropriate environment, providing adequate equipment, recruiting appropriate manpower, and clarifying the expectations are some strategies for improving patient handover conditions.

## Introduction

Improving patient handover is currently considered as a patient safety goal by “The Joint Commission Accreditation of Health Care” since 2006 and one of the top five world health organization (WHO) priorities (1). 

Patient handover is described as sharing information, authority and answerability between individuals and teams in the health care system (2). It plays an important role in maintaining continuity of care and also a key role in teaching new students, promoting team cohesion, emotional support, socialization, maintaining social order, patient safety, and mistake detection (3, 4).

The busy, overcrowded, noisy and distracting environments of an emergency department (ED) coupled with time pressure, make the handover of the patient susceptible to data loss, misinformation and high rates of mistake (5). The primary phase of care in ED, including triage, resuscitation and intervention, is especially error-prone. During this time-pressured period, important decisions are being made based mainly on information provided by paramedics for the ED members (2). The transfer of care between paramedics and ED members includes a handover of information, which contains demographic details, the event preceding the call, their findings, treatment and details of patient’s medical history (6). This handover is usually reported verbally, but it must be documented to provide a formal record of care for quality assurance purposes and patient safety (7). However, there is often little time to document extensive information resulting in dependence on memory when providing verbal handover. Nevertheless, cultural, linguistic, and social differences between paramedics and ED members may increase the potential for conflict or misunderstanding (4). 

The most recent review on handover concluded that the quality of research on the topic is really primitive, and there are not yet enough studies to guide evidenced-based handover practices and little is known about how effectively data is transferred from paramedics to ED members who receive the patients (1, 8-10) . A study by Evan et al. (2010) showed that only 34% of information verbalized by paramedics was recalled by emergency nurses (11). Also, a study of handover in a simulated situation showed that 18% of information transfer to other personnel was inaccurate. Furthermore, there is also evidence that the documentation of information handed over by paramedics to the ED members is not completely accurate and only 67% of the information is accurate (2). 

Since effective handover is critical to achieving the optimum management of all patients (5), communication errors are costly both for the patient and the economy (11), and transfer of care among providers has been identified as a major source of medical error (4), the present study aimed to explore the perspectives of paramedics and ED members regarding patient handover. 

## Methods

***Study design and setting***

A qualitative design with inductive content analysis was used for data collection and analysis of patient handover perspectives among paramedics and ED members of Valiasr Hospital, Fasa, during 2015.

Fasa is a city in Fars province, Iran, with a population of about 210 thousand people, having two hospitals with three urban EMS units and ten road EMS bases. Fasa is located in a geographic place through which a transit road passes and increases the probability of road accidents. In this city, paramedics are sent on an average of 15000 missions each year, 65% of which is related to internal medicine such as heart disease and 35% are related to accidents and trauma.

***Ethical considerations***

 This study was approved by the Ethics Committee and Research Council of Fasa University of Medical Sciences. Before each interview, the participants were informed about the study objectives and procedures by one of the research team members and were told that their participation was voluntary. Then, they were required to sign written informed consents for taking part in the research. Besides, they were reassured that they could quit the study any time they wished. Confidentiality was also ensured by considering anonymity. 

***Participants***

The study participants were selected through purposeful sampling. This sampling method is generally used in qualitative researches and helps select information-rich participants (12). The researchers interviewed the paramedics and ED members who had rich experience and information about this phenomenon. The inclusion criteria of the study was having at least two years of clinical experiences, and willing to participate in the study. Overall, eleven nurses and fourteen paramedics were selected.

***Data gathering***

Data were collected through semi-structured interviews using guide comprising probing questions in 2015 by a nurse qualified in qualitative research. The interviews were performed by one of the researchers who was experienced in qualitative researches. Further supports were also provided by other team members who were experienced in emergency nursing. The interviews were audio recorded and transcribed verbatim after each session. In total, twenty-five in-depth interviews were conducted each lasting 60-90 minutes. It should be noted that the interviews were conducted in places free from distractions and at times and locations that were most suitable for the participants. Prior to the interviews, the researchers established rapport with the participants. Then, interviews began with general questions and moved toward more detailed inquiries depending on the participants’ responses. Major interview questions were as follows: “Can you describe today’s handover?”, “What is your opinion about patient handover in ED?”, “How is patient handover in your department?” and “What is it like?”. At the end of the interviews, the researchers thanked the participants for their time and asked them if there was anything they would like to add. The interviews continued until reaching data saturation point where no new information was gained.

***Data analysis***

Conventional content analysis proposed by Graneheim and Lundman was used to analyze the data (13). After each interview, its contents were documented by the research team immediately. Then, the data were read several times to gain a general understanding of the participants’ statements in line with the study objectives. After that, the research team extracted meaning units or initial codes, which were eventually merged and categorized according to similarities and differences. MAXqda2 software 10.0 R250412 was used for data analysis. In the end, the final codes, including their defining properties and their relationships with each other, were reviewed in order to reach a consensus regarding the central, unifying themes emerging from the data. 

***Rigor***

The procedures that were used to improve data trustworthiness were as follows: coding and categories were sent back to the participants for possible revisions. A team-based approach to analyze data was established to check the credibility. This showed a good level of agreement in interpretation and some disagreements were resolved through discussion. Prolonged engagement, varied experiences, and peer checking were other strategies employed for improving the trustworthiness of the study (12-14) .

## Results

25 paramedics and ED members with the mean age of 37.2 ± 3.5 (18 - 60) years and mean job experience of 8.2 ± 3.8 (2 – 12) years participated in the study (80% male). The baseline characteristics of participants are listed in table 1. 

One main theme, two major categories and four subcategories were generated through data analysis (table 2). All in all, analysis of multiple sources of data indicated that patient handover is a sophisticated process, which is an encounter between two separate peninsulas with different extrinsic and intrinsic factors. 

***Different Extrinsic factors:***

*1) Different Environment*

The first subcategory emerging from data was “*Different environment*” as one of the perspectives of paramedics and ED members regarding patient handover. Almost all of the participants mentioned lack of a good environment for transferring patient information to others as a problem. They highlighted the importance of revising the architecture for improving communication during patient handover. 

In this regard, a paramedic said: *“In my opinion, the major weakness of patient handover is space and building. Emergency departments and triage are like a “crossroad”. It is very crowded and we don’t have enough concentration for reporting”.*

Or another nurse added: “*We cannot keep patient privacy during reporting. Anybody can come near the patient and listen to his/her history. It is like a sidewalk here”.*

Or another nurse said: * “Yesterday, the triage was very crowded. You could hear a lot of noises such as screaming, shouting,* *using* *obscene language, anything except reporting of patient handover. There were approximately ten people near a patient’s stretcher”. *

*2) Different Equipment*

The second category that was extracted from the data is “*different* *equipment*”. The participants strongly believed that different equipment of ambulance and ED is another challenge that increases waste of time. The following excerpt from a paramedic illustrates that different equipment is another problem. 

*“This is my greatest pain, the equipment of our ambulance is different from that of the hospital such as stretcher, backboard or other equipment, so we should go to a storage room and search for the equipment, and this process will waste our time and lengthen our mission”.*

Another nurse expressed a similar perception. *“One of our challenges with paramedics is about equipment, our equipment in hospital is not appropriate for ambulance, so in some situations, we have to move the patient with a broken leg fixed in order to give their equipment back. This will be very dangerous for that patient”. *

***Different Intrinsic factors:***

*1) Different Manpower*

The third subcategory emerging from the data was “*different* *manpower*”. The participants acknowledged that recruiting appropriate manpower is very important. These participants felt that one of the challenges of patient handover is different education of manpower or lack of a qualified one. The following narrative statement described this subcategory:

*“We have some colleagues that have different educational backgrounds such as Surgery Technology or Anesthesiology. They really didn’t know anything about patient handling in emergency situations, so in patient handover, they cannot transfer accurate information to others”.*

For example another nurse mentioned: 

“*Communication during patient handover with paramedics is hard; I think we have different experiences and languages that interfere with the clarity of speech”.*

Also regarding this subcategory a participant stated that:

*“Lack of qualified security and service members in this ward is another problem; they should be present during patient handover and organize the situation”.*

*2) Different expectations*

The last category that was extracted from data was “*Different expectations*”. They mostly mentioned that during patient handover each system had different expectations from us. In this regard, a paramedic reported: “*Nurses asked us to transfer the patient completely and help them in some emergency situation to stabilize the patients; on the other hand, our managers want us to leave the hospital as soon as possible, in less than 10 minutes and come back to our units and become ready for the next mission”*.

The following statement from a paramedic shows the underpinnings of this theme:

*“In some situations, managing the patient in emergencies and in the ambulance is too hard, but the nurses in emergency ward expect us to transfer the patient completely during patient handover”.*

## Discussion

The aim of this study was to explore the perspectives of paramedics and ED members on patient handover. They believed that their handover is a sophisticated process, which is an encounter between two separate peninsulas with different extrinsic and intrinsic factors such as different environments, different equipment, different manpower, and different expectations.

 Our results is in congruence with Meisel et al. (2015) who said that patient handover between paramedics and ED members has unique challenges, because they have different clinical duties and professional culture and largely non-overlapping sites of work, leading to potential communication errors and teamwork conflicts that could be costly, especially for ill patients in the context of care (15). 

Our study showed that patient handover did not occur in an appropriate environment. Since patient handover takes place in a time-limited period and paramedics are often required to transfer sophisticated problems to multi-professional members, this transition is highly susceptible to information loss. In this regard, Mcdonagh et al. (2013) conducted a study “Clinical Handover at the Emergency Department - A two way process” and found that handover can take place anywhere in the department and they recommend a specific location for patient handover (16). Moreover, Manser et al. (2011) highlighted that environmental factors will undermine the effectiveness of inter-professional communication across a variety of settings (17). In this regard, Sabet Sarvestani et al. (2015) conducted a study in pediatric department and explored the challenges of nursing handover. They found that there was not a quiet room for nursing handover, which could lead to many interruptions and decrease the quality and accuracy of handover. They recommend allocating a place far from interruptions for patient handover to improve patient data confidentiality and privacy (18). As Bost et al. (2012) said ED is a chaotic and complex environment, which can result in inaccurate transfer and loss of information during handover process. The design and building quality (architecture) of an ED has principal influence on the health, safety and satisfaction of personnel and patients as well as efficiency of care. So, architectural planning and organization of ED based on the standards and allocation of a special place for handover is very important (19). On a number of occasions the nursing staff mentioned the adverse effect of a noisy ED on cognitive tasks and their ability to hear handover by paramedics. Noises reduce performance of complex tasks and increase risk of error and staff burnout in the critical care setting. Moreover, Evans et al. (2010) suggested teaching effective listening skills to trauma team in health care systems (2). 

Another subcategory that emerged from our data was “*different equipment*”. As mentioned before, having a storage to facilitate re-stocking of ambulances that require rapid turnover is a necessity for each standard emergency department (20). Also, Day et al. (2010) in their article pointed to this matter and said that it is mandatory for each hospital to have a protocol for intra-hospital transport. This protocol should address different parts of handover such as pre-transport coordination, necessary equipment, monitoring process, ways of documentation, and transport personnel/training. Availability of equipment in the same size and shape in hospital is recommended to decrease paramedics’ waste of time after patient handover (21). 

**Table 1:** Demographic characteristics of the participants

**Characteristic**	Number (%)
**Sex **	
Male	20 (80)
Female	5 (20)
**Age (year)**	** Mean **
18-25	7 (28)
26-30	5 (20)
31-40	10 (40)
41-50	2 (8)
51-60	1 (4)
**Marital status **	
Married	23 (92)
Single	2 (8)
**Specialty **	
Paramedic	14 (56)
Nurse	11 (44)
**Job experience (years)**	
< 5	5 (20)
5-10	15 (60)
>10 y	5 (20)

**Table 2:** Main category and related subcategories extracted from participant perspective

**Main theme**	**Categories**	**Subcategories**
Two separate peninsulas	Different Extrinsic factors	Different environment
		Different equipment
	Different Intrinsic Factors	Different manpower
		Different expectation

As another aspect of our findings, different manpower was another subcategory that explained the perspective of paramedics and ED members regarding patient handover. Although ambulance services play a key role in health care system, there are no good strategies for selection of these groups and little attention has been paid to their improvement. A change in the educational process is necessary to monitor the recruitment of paramedic workforce overtime (22). Also, Woolard et al. (2006) said that recently the number of emergency ambulance services has increased markedly and successful development of paramedics’ role needs a shift from vocational training to educational institution (23). Meisel et al. (2015) suggested some solutions for improving patient handover such as increasing EMS interactions with emergency physicians and standardizing handover processes (15). However, standardization by itself is not enough. Multidisciplinary training in combination with introduction of a handover tool that requires minimal information is recommended (1). 

The perspectives of our participants highlighted the importance of the presence of security staff during patient handover. A College for Emergency Medicine published a guideline for emergency department design and pointed that in all emergency departments, the proximity of security staff, preferably visible all the time, is important (20). Jensen et al. (2013) said that handover is a conversation between the professionals that might improve empathy, equinity and common ground and is clearly not just an exchange of information. Having different values, language and hierarchies hinder the efficacy of patient handover, they suggested a change in work-related culture from individual health professional culture to developing a common multi-professional team culture (1). In a study conducted by Fairbanks et al. (2007) it was confirmed that communication errors are evident between paramedics and ED members who would ultimately take care of the patient during their ED stay (24). 

The last subcategory was “*different expectations*”. An ED is a complex workplace with a set of interdependent facilities involving many different professionals serving every single patient (7). Bruce et al. (2005) found that there would be a role conflict between paramedics and ED members that can be confounding during patient handover, especially when paramedics assume more responsibility than expected (7). Since the role of paramedics has recently increased in our new health care system, the rights and laws will be required to support paramedics in undertaking an extended role (23). In this regard, as it is advocated by many, including WHO, Carter et al. (2009) and Delupis et al. (2014) suggested designing an appropriate method of transmitting and receiving data and using a standardized approach to handover communication in ED (8, 25). 

Daily activities can cause a high level of stress, especially at the time of high patient turnover. For this reason, it is imperative that clear, consistent and concise communication be undertaken to minimize the risk of incidence of adverse events. The findings of the present study challenge managers to develop new strategies that can improve patient handovers, which in turn can facilitate changes that increase the level of job satisfaction among ED members and paramedics. As a result, these can lead to a higher level of patient safety with a higher quality of care. Patient handover is a skill that requires education and practice, so in this regard, in-service education is a priority and highly recommended. Since improving handover practices mainly depends on the context, which is completely different in each organization due to different substructures, facilities, aims, priority and economic situation, action research is recommended to face the challenges of real-world problems.

***Limitations***

Generalizing our findings to other clinical settings is a limitation as our study took place in one department. Thus, it is recommended that further studies be conducted in other departments. Furthermore, although we did twenty-five interviews, we may have missed other perspectives. Despite these limitations, our findings captured a good view of the current situation for provision of a foundation to plan and implement appropriate change.

## Conclusions

In general, our data analysis indicated that designing an appropriate environment, recruiting appropriate manpower, providing adequate equipment and clarifying the expectations are some strategies for improvement of patient handover between EMS and ED personnel. 
